# Effects of an Eating Pattern Including Colorful Fruits and Vegetables on Management of Gestational Diabetes: A Randomized Controlled Trial

**DOI:** 10.3390/nu15163624

**Published:** 2023-08-18

**Authors:** Kataryna Jaworsky, Pamela DeVillez, James M. Alexander, Arpita Basu

**Affiliations:** 1 Kirk Kerkorian School of Medicine, University of Nevada, Las Vegas, NV 89154, USA; jawork1@unlv.nevada.edu; 2 Department of Kinesiology and Nutrition Sciences, School of Integrated Health Sciences, University of Nevada, Las Vegas, NV 89154, USA; pdeville@unlv.nevada.edu; 3 Department of Obstetrics and Gynecology, UNR School of Medicine, University of Nevada, Reno, NV 89557, USA; jamesalexander@unr.edu

**Keywords:** gestational diabetes mellitus, lifestyle modification, diet, hyperglycemia, flavonoids, fiber, antioxidants

## Abstract

Gestational diabetes mellitus (GDM), defined as abnormal glucose tolerance that presents during the second and third trimesters of pregnancy, is a growing issue in the United States and worldwide. If left untreated or poorly controlled, GDM can result in numerous consequences for both the mother and the fetus; thus, it is imperative that different avenues of management for GDM be explored. There is a paucity of studies that examine how lifestyle changes, including dietary and physical activity, affect management of GDM. We examined how counseling on lifestyle changes can affect cardiometabolic risks in women with GDM. We conducted a 12-week randomized controlled trial based on behavioral counseling in which women with GDM (N = 38) were randomized into either a nutrition education (control) (N = 18) group or nutrition intervention (N = 20) group. The nutrition education group were given dietary counseling regarding healthy dietary choices based on USDA guidelines, while the nutrition intervention group were instructed to consume a total of one cup of whole berries and one cup of leafy vegetables daily along with performing postprandial exercise (walking). Blood samples, anthropometric measures, and dietary and physical activity data, recorded in daily food and activity logs, were collected at baseline and at the end of the study and compared between the two groups. Dietary counseling on supplementation with whole berries and leafy vegetables resulted in increased fiber intake, increased antioxidant intake and total serum antioxidant capacity, improved random blood glucose, decreased serum IL-6, and improved HDL cholesterol versus the control group (all *p* < 0.05). These results highlight that whole berry and leafy vegetable supplementation-based dietary counseling can improve the metabolic pathways involved in gestational diabetes pathogenesis and prognosis. These functional foods must be recommended in the management of pregnancies affected by GDM.

## 1. Introduction

Gestational diabetes mellitus (GDM), characterized as abnormal glucose tolerance that first presents during pregnancy in the second or third trimester, is a growing problem in the United States, affecting up to 10% of pregnancies each year [1,2]. Often, GDM is a result of insufficient pancreatic function of the mother to overcome the increased insulin resistance that is common during pregnancy, thus resulting in poor glycemic control [3]. The pregnant woman therefore experiences hyperglycemia, which can have consequences for both the mother and the fetus. In the mother, there is a higher risk of gestational hypertension and preeclampsia, as well as delivery complications [2]. Furthermore, GDM can adversely affect fetal growth and development, and maternal hyperglycemia and insulin resistance can influence the fetus’s pancreatic function in utero and ultimately result in hypoglycemia at birth [4]. Birth complications may also arise, including macrosomia and shoulder dystocia of the child, which may result in the mother having a cesarean section delivery to prevent any complications [5]. A child born to a mother with GDM during pregnancy is also at heightened risk of developing type 2 diabetes mellitus later in life [6]. Management of GDM is of urgent public health importance, and yet there are few research-based dietary and lifestyle interventions that have been elucidated that have a direct effect on glycemic control in these patients. 

The increased prevalence of GDM, along with the growing obesity epidemic in our country, which increases a pregnant woman’s risk of developing GDM, highlights the need for research towards mitigating the effects of GDM [7,8]. The current mainstay of treatment of gestational diabetes is glycemic control. Oftentimes, insulin and metformin are prescribed to patients at the time of diagnosis, but lifestyle modifications are also included in the treatment plan. Lifestyle modifications, including alterations in diet and physical activity levels, can help promote better insulin usage and therefore improve glycemic control via increased insulin sensitivity and glucose uptake following exercise [9]. Moreover, lifestyle modifications are often easier, cheaper, and have fewer side effects as compared to medication alone and lead to significant improvement in glycemic control even with moderate modifications to the diet. 

Observational studies have been conducted, and they demonstrate a correlation between dietary modifications and decreased risk of developing GDM [10,11]. It has been shown that diets characterized as low in carbohydrates and high in vegetable protein have been associated with a decreased risk of GDM development [10]. Women who consumed more fresh fruits during their pregnancy were also shown to have a decreased risk for the development of GDM [12]. These observational studies have shown a link between increased fruit and vegetable intake and GDM risk and progression [13,14]. Other studies have demonstrated that, in healthy non-pregnant subjects, increased whole berry consumption resulted in decreased postprandial glucose and increased insulin levels, suggesting improved glycemic control immediately following meals [15]. Furthermore, the addition of raw vegetables to the diets of adults with type 2 diabetes directly improved glycemic control in patients in a randomized controlled trial (RCT) [16]. This is likely because fruits and vegetables are high in metabolically active polyphenolic flavonoids that positively modulate the insulin signaling pathway, which results in reduced insulin resistance [17]. This reduced insulin resistance promotes improved glucose metabolism, and thus minimizes the hyperglycemic state often observed in diabetic patients. Given that both GDM and type 2 diabetes have similar mechanisms of insulin resistance, it can then be postulated that the mechanism of improved glycemic control with dietary active polyphenolic flavonoids found in raw fruits and vegetables can be beneficial in patients with GDM as well. 

There is also a paucity of studies reported that show a direct correlation between altered diet with increased physical activity and prognosis of GDM in already diagnosed women. Most studies, as discussed, are observational studies in which the outcome measured is risk of developing GDM. While prevention of GDM is important, it is imperative that further research be conducted to determine dietary alterations that can have a direct effect on glycemic control, and ultimately prognosis, of women with GDM. Thus, we conducted a RCT in which women with GDM were instructed to increase the consumption of berries and green leafy vegetables, as well as increase postprandial physical activity. We hypothesized that these lifestyle interventions will improve cardiometabolic profiles in these GDM patients.

## 2. Methods

This was a randomized controlled trial conducted at the UNLV School of Medicine Maternal Fetal Medicine Clinic at Sunrise Hospital in Las Vegas, Nevada. The clinical trial was approved by the ethics committee at UNLV (IRB# 1610677). All participants provided written informed consent upon enrollment in the trial. The trial is registered at Clinicaltrials.gov website (NCT04861324).

### 2.1. Study Criteria and Protocol

Adult women with a GDM diagnosis were enrolled in the study and randomized into one of two treatment groups to analyze the effects of dietary and lifestyle modification on cardiometabolic profiles in the mother. The inclusion criteria for this study were pregnant women between the ages of 18 to 45, were currently in their 24–28 weeks of gestation during the initial screening appointment, had high blood glucose (diagnosed either via 1 h or 3 h glucose tolerance test), history of diabetes (either GDM in a prior pregnancy and/or type 2 diabetes) prior to pregnancy, and current singleton pregnancy. Exclusion criteria included allergies to berries and/or leafy vegetables, current tobacco or alcohol use, current lactation, current multiple gestation pregnancy, or unwillingness to provide written informed consent. Upon providing informed consent and enrolling in the trial, participants were screened at their 24–28-week clinic appointment, where a screening questionnaire was completed regarding dietary habits, anthropometric measurements were obtained, and a blood draw was completed for baseline metabolic parameters prior to the initiation of the intervention. Patients were then randomized into one of two treatment groups, detailed below, and followed the recommended dietary and lifestyle modifications until delivery. Patients received lifestyle counseling based on their assigned treatment group biweekly via either telemedicine or telephone appointments. Patients were also encouraged to keep a food log for any three days each week. At the patient’s 36–40-week gestation clinic appointment, anthropometric measurements were determined, another blood sample was obtained for repeat metabolic parameter measurements, and food diaries were collected to be analyzed. Patients were encouraged to continue to follow the assigned interventions until delivery.

In total, 38 patients were enrolled into this study and randomized into a treatment group, with 18 being assigned to the control nutrition education group and 20 being assigned to the nutrition intervention group.

### 2.2. Intervention and Control Groups

Patients enrolled in the study were randomized into either a control nutrition education or a nutrition intervention group. Each participant was provided handouts with recommendations for their specific treatment group and were provided biweekly counseling appointments as detailed above. For the control group, termed the ‘nutrition education (NE)’, a registered dietician provided counseling based on the USDA Choose My Plate guidelines. The intervention group, termed the ‘nutrition intervention (NI)’, were given specific dietary counseling to enact during their enrollment in the study. In this group, patients were provided educational materials to incorporate specific dietary changes. Patients were instructed to consume ½ cup of berries as a mid-morning snack and another ½ cup of berries 6–8 h later and to consume one cup of leafy greens for lunch and/or dinner. Instructions were given to consume the berries and leafy greens of their selection in their whole fruit and vegetable form, meaning it was advised against blending or juicing the fruits and vegetables. Participants in this intervention group were instructed to maintain a food diary and record what fruits and vegetables were consumed at least three days each week. NI participants were also instructed to perform physical activity after meals, mainly via 15 to 30 min walks, and to maintain a journal of any physical activity performed during the duration of the study. 

### 2.3. Biochemical Analyses

Women were screened between 24 and 28 weeks of gestation using the two-step criteria: first step, non-fasting 50 g oral glucose challenge test (GCT); then, if positive: second step, 100 g GCT administered in the fasting state. GDM diagnosis was confirmed if a participant exceeded threshold levels in both criteria. This screening protocol is widely used in US institutions and recommended by the American College of Obstetrics and Gynecology (ACOG) [18]. Freshly drawn blood samples were sent to Quest Diagnostics (Las Vegas, NV, USA) for analyses of blood glucose, lipids, C-reactive protein, and glycosylated hemoglobin. Serum interleukin-6 and total antioxidant capacity were measured using ELISA assays according to the manufacturer’s protocol (R&D Systems). 

### 2.4. Dietary Analyses

All study participants were asked to maintain a 24 h food recall for three days/week, which they submitted to the RD during their biweekly visits and discussed any changes they made in their habitual diet. Dietary analyses were conducted by the study RD or a trained dietetic assistant using ESHA’s Food Processor^®^ Nutrition Analysis software for energy, nutrients, and food group intakes for each participant. Total flavonoid intake was determined using the USDA flavonoid database [19]. Each participant was asked whether they exercised regularly and for how many minutes. To be classed as “physically active”, participants needed to perform habitual exercises such as regular walks, jogging, total body workout at a gym, swimming, yoga, Pilates, fitness, exercise ball workouts, or home gymnastics as recommended by ACOG [20].

### 2.5. Statistical Analyses

For each measure, descriptive statistics were examined to identify outliers: none were found. For baseline demographics and characteristics, continuous variables were expressed as means ± SDs and discrete variables as percentages. We employed a 2 × two-factor repeated-measures ANOVA (MIXED procedure; group: NE, NI; time: baseline and end) to examine the main effects of group, time, and whether overall changes in time differed between the two groups (interaction). Baseline values were included as covariates for each outcome variable. From our previous dietary intervention study in women at risk for GDM, we expected a mean ± SD difference in maternal blood glucose levels of 8.5 ± 3.6 mg/dL with a sample size of 12 in each group within eight weeks in the present study to achieve 80% power at an α level of 0.05 [21]. All analyses were conducted using SPSS (version 26.0).

## 3. Results

### 3.1. Baseline Characteristics

Table 1 outlines the baseline characteristics of the 38 women who completed the study, all of whom were self-reported Hispanic women. None of the variables are significantly different between the two groups, evidenced by all *p*-values being greater than 0.05, which highlights that the two groups were statistically similar at the beginning of the trial in anthropometric, metabolic, and dietary parameters. This allows for better comparison of the primary endpoints and ensures that any significant results after the nutrition intervention were likely due to the intervention itself and not confounded by baseline differences between the groups. 

### 3.2. Dietary Intake

Table 2 presents the mean endpoint values of dietary variables in both the control and the nutrition intervention groups. As expected, the nutrition intervention group consumed significantly higher amounts of vegetables and fruits, quantified as a percent of the recommended dietary allowance (mean ± SD: 57 ± 10 and 68 ± 15, respectively), as compared to the nutrition education group (44 ± 19 and 50 ± 12, respectively). The nutrition intervention group also demonstrated a significantly higher average dietary fiber intake (23 ± 7 g/day) as compared to the control group (15 ± 6 g/day). Finally, mean total flavonoid intake was greater in the nutrition intervention group when compared to the total flavonoid intake of the control group (62 ± 16 mg/day and 45 ± 14 mg/day, respectively) (all *p* < 0.05). 

### 3.3. Anthropometric and Cardiometabolic Markers 

Table 3 presents the anthropometric data, none of which were significantly different between the intervention and control groups, as well as cardiometabolic parameters measured at the end of each group’s assigned intervention. Random blood glucose was significantly lower in the nutrition intervention group at 112 ± 14 mg/dL by the end of the intervention period as compared to the control group’s level of 128 ± 24 mg/dL. HDL-cholesterol was significantly higher in the intervention group (51 ± 7 mg/dL) as compared to the control group (43 ± 8 mg/dL). Average serum IL-6 levels were lower in the nutrition intervention group than in the nutrition education group (26 ± 13.8 and 34.6 ± 12.2, respectively). Finally, total serum antioxidant capacity was significantly increased in the nutrition intervention group, 6.3 ± 3.8 µmol/L, compared to levels of the control group, 3.8 ± 3.5 µmol/L (all *p* < 0.05).

### 3.4. Physical Activity

In addition to the various cardiometabolic and anthropometric data examined during this clinical trial, physical activity was also measured before and after intervention. The nutrition intervention group was asked to begin incorporating physical activity after meals, mainly via 15 to 30 min walks, and to maintain a journal of any physical activity performed during the duration of the study. Physical activity at baseline was not statistically significantly different between the groups, with the control education group reporting an average 23 ± 12 min daily physical activity and the nutrition intervention group reporting 17 ± 11 min of daily physical activity (*p* = 0.43, Table 1). After the 12-week intervention period, the control group had an average of 30 ± 12 min of daily physical activity and the intervention group had an average of 25 ± 13 min of daily physical activity, which again was not statistically significant (*p* = 0.24, Table 3). 

## 4. Discussion

In our randomized controlled trial of women diagnosed with GDM, we demonstrated that dietary counseling focused on the addition of whole berries and leafy vegetables resulted in multiple improvements in dietary profiles and markers of cardiometabolic risks in the mother. Importantly, the baseline characteristics of each treatment group were not significantly different from one another. Additionally, daily exercise did not significantly differ between the control and intervention groups after the 12-week intervention period despite the intervention group being instructed to increase their daily postprandial physical activity. Therefore, it can be inferred that all the significant differences elucidated in this trial are due to the dietary interventions that the intervention group were instructed to begin and follow. The implementation of the USDA dietary guidelines-based nutrition education is quite ethical as a control treatment for these high-risk women as part of standard prenatal care. Thus, the significant outcomes observed in the nutrition intervention group clearly shows the effectiveness of evidence-based food group-specific counseling in improving cardiometabolic profiles in GDM. 

Diets rich in fruits and vegetables have been shown to improve gestational diabetes risks, as well as its management in observational studies and RCTs [22,23]. In our study, at the end of the 12-week dietary counseling, the intervention group met 68 ± 15 percent of the recommended dietary allowance (RDA) of fruit intake, while the control group had 50 ± 12 percent of the RDA of fruit met; the intervention group also met a 57 ± 10 percent RDA of vegetables as compared to the control group with 44 ± 19 percent RDA met. These results provide evidence of compliance to the dietary recommendations in both groups. As a result, the intervention group had an average of 23 ± 7 g/day daily fiber intake whereas the education group had an average daily fiber intake of 15 ± 6 g/day. It is well understood that fruits and vegetables are rich sources of fiber in the daily diet [24]. Zhang et al. (2021) reported in their randomized controlled trial that the addition of dietary fiber during the second trimester of pregnancy dramatically decreased blood glucose levels in patients with GDM [25]. This highlights the importance of increased dietary fiber during the second trimester, which is when most patients are diagnosed with GDM. The meta-analysis conducted by Sun et al. (2022) summarized the findings of multiple clinical trials that showed dietary fiber intervention reduced fasting glucose, two-hour postprandial glucose, glycosylated hemoglobin (HbA1c), total cholesterol, triglycerides, and LDL cholesterol, all of which are important biochemical markers of GDM prognosis [26]. 

In addition, total dietary flavonoid intake was also significantly different between the two groups, with the nutrition intervention group consuming an average 62 ± 16 mg/day total flavonoid intake and the nutrition education group consuming an average 45 ± 14 mg/day total intake. Flavonoids are metabolically active compounds found in a wide range of plant-based foods, including berries and vegetables, that are known for their anti-inflammatory and antioxidant functions. Specifically, flavonoids inhibit the synthesis and function of various pro-inflammatory mediators via multiple mechanisms, one of which is the inhibition of the transcription factor NF-kappaB; however, large concentrations of flavonoids are needed to exert a physiological effect [27]. Thus, increasing the intake of flavonoids, via increased berry and vegetable intake, can result in improved anti-inflammatory functions in the system. While there are observational studies, such as the one by Gao et al. (2021), that demonstrate higher dietary intake of flavonoids from fruits is associated with lower risk of GDM development [28], there is a lack of clinical trials examining the effects of flavonoids on GDM management, not risk. Al Duhaidahawi et al. (2021) summarized research in type 2 diabetes and concluded that flavonoids help regulate glucose metabolism directly but has yet to be confirmed in patients already diagnosed with GDM [29]. 

As discussed earlier, most studies on dietary habits and GDM often analyze the dietary habits and the risk of developing GDM; few studies investigate these same dietary changes during pregnancy in an already diagnosed GDM patient. The observational study by Sun et al. (2022) demonstrates that fruit intake in the first trimester directly affects blood glucose metabolism and results in a decreased fasting blood glucose level as well as a decreased risk of developing GDM in the second trimester [30]. Moreover, the randomized control trial by Sahariah et al. (2016) illustrated that a daily snack with leafy vegetables, fruits, and milk before and during pregnancy was associated with lower 2 h glucose concentrations and ultimately had a protective affect against GDM development [31]. While these studies highlight important dietary changes that can be made for GDM prevention, these studies are just a subset of those that focus on prevention of GDM and not management of GDM, of which studies are lacking. Our RCT has demonstrated that supplementation of whole berries and leafy vegetables during active GDM results in overall lower blood glucose levels, and given the randomization, we are able to infer that the decreased blood glucose, and thus better GDM control, is a direct effect of the dietary changes made. Interestingly, despite the significant decrease in random blood glucose due to the increased intake of whole berries and leafy vegetables, the difference in HbA1c between the two treatment groups was not significant. This is likely because HbA1c is an average of the glycosylated hemoglobin over 90 days as part of the red blood cell lifespan, and the intervention time from enrollment (24–28 weeks gestation) to delivery (36+ weeks gestation) was probably not long enough to affect the average changes in HbA1c. Nevertheless, the decrease in random (postprandial) blood glucose is clinically meaningful as this has been independently associated with both future development of type 2 diabetes mellitus and future CVD events in diabetes [32,33,34]. 

The average HDL-cholesterol (HDL-C) in the intervention group was significantly higher at study end at 51 ± 7 mg/dL as compared to the control group’s HDL-cholesterol at 43 ± 8 mg/dL. HDL-cholesterol, often referred to as the ‘good cholesterol’, is important for transport of cholesterol back to the liver for processing, thus lowering total circulating cholesterol [35]. Low levels of HDL-C are also implicated in endothelial dysfunction, one of the key mechanisms behind the development of GDM in at risk patients [35]. A previous randomized controlled trial conducted by Basu et al. (2021) found that dietary blueberry and soluble fiber supplementation did not result in improved or worsened HDL-C as compared to the control group, but these patients were simply at risk for GDM and were not yet diagnosed [21]. Again, there is a paucity of research looking into dietary changes in patients already diagnosed with GDM and the effects that dietary supplementation has on metabolic and biochemical biomarkers such as cholesterol levels. Flavonoids, however, have been implicated in the improvement of HDL-C levels in type 2 diabetic patients, specifically in the clinical trial by Basu et al. (2015) that demonstrated increased dietary cocoa intake, a rich source of flavonoids, resulted in increased postprandial HDL-C levels [36]. The nutrition intervention group in our study had significantly higher total flavonoid intake at the end of the study as compared to the nutrition education group, and we can postulate that the increased flavonoid intake was responsible for at least some of the improvement in HDL-C in the intervention group. Flavonoids are also found in high quantities in various fruits, including whole berries, and have been implicated in the improvement of various cardiometabolic markers in diabetic patients. Curtis et al. (2022) demonstrated that intake of blueberries, a fruit high in anthocyanins, resulted in improvement of numerous cardiometabolic markers including improved HDL-C levels as well as improved postprandial glucose, total cholesterol, and insulin levels in patients with metabolic syndrome [37]. However, as previously mentioned, studies focusing specifically on flavonoids and their impact on GDM management are lacking. Our study supports the conclusion that dietary sources rich in flavonoids, such as whole berries, can be recommended to GDM patients for management of their condition. 

The nutrition intervention group had a significantly lower average serum IL-6 at 26 ± 3.8 pg/mL compared to the 34.6 ± 12.2 pg/mL average in the control group in our study. IL-6 is a cytokine that has been implicated in the pathogenesis of GDM via its pro-inflammatory impact on endothelium dysfunction and insulin resistance, suggesting that high levels of IL-6 are correlated with increased insulin resistance, leading to potentially higher blood glucose and worsening prognosis of GDM [38]. Numerous observational studies have elucidated that IL-6 is elevated in GDM affected pregnancies, such as the study by Zhang et al. (2017), but no published studies examine the modulation of IL-6 in already diagnosed GDM patients in relation to dietary changes [39]. Thus, our study demonstrating that IL-6 levels were significantly decreased in the intervention group after the dietary and lifestyle interventions suggests that the insulin resistant state was also decreased in GDM patients. This finding, together with the decreased blood glucose seen in the intervention group, illustrates that the fruits and vegetables directly contribute to improvement of GDM condition.

Finally, the increased intake of berries and leafy vegetables resulted in a significant increase of total serum antioxidant capacity in the nutrition intervention group at 6.3 ± 3.8 µmol/L while the nutrition education group’s total serum antioxidant capacity was 3.8 ± 3.5 µmol/L. The case–control study conducted by Parast et al. (2017) demonstrates that the total antioxidant capacity in women diagnosed with GDM was significantly lower than healthy pregnant women [40]. To our knowledge, there are no studies examining the effects of whole berry and vegetable intake on antioxidant capacity in GDM. Duthie et al. (2018) examined the effect of increased whole fruits and vegetables, along with fruit juices, in healthy adults who self-reported low fruit intake on antioxidant capacity and found no significant changes in antioxidant capacity with the increased fruits and vegetables [41]. However, these adults were healthy and not in an inflammatory state, unlike those with gestational diabetes. Our study demonstrates that dietary counseling emphasizing addition of whole berries and leafy vegetables to the diets of GDM patients results in significantly higher serum antioxidant capacity, which has direct effects in decreasing oxidative stress underlying the pathogenesis of gestational diabetes [42].

The nutrition intervention group was also instructed to increase their postprandial physical activity via 15–30 min brisk walks after meals. Despite this guidance, there was no significant difference in the minutes per day spent walking, or any other physical activity, between the nutrition education and nutrition intervention groups. Although our study demonstrated that verbal instruction was not potent enough to result in a significant difference in postprandial physical activity in GDM patients, there have been RCTs that demonstrate the effectiveness of physical activity both in reducing GDM risk and for controlling active GDM. Hayashi et al. (2018) demonstrated in their RCT that an increase in walking during pregnancy to greater than 6000 steps per day was effective at lowering random glucose levels in patients with GDM [43]. The systematic review conducted by Laredo-Aguilera et al. (2020) highlights that any type of physical activity, whether it be aerobic, resistance, or a combination, is effective for the control of glucose, HbA1c, and insulin levels in pregnant GDM patients [44]. Finally, the meta-analysis conducted by Huang et al. (2020) showed that increases in aerobic exercise, which includes walking, reduced fasting blood glucose, postprandial blood glucose, and HbA1c levels in patients with GDM [45]. Despite our study not demonstrating a significant relationship between physical activity and GDM control in the nutrition intervention group, the multiple studies documenting improved outcomes with increased exercise provide evidence to support continued recommendations of increased physical activity for glycemic control in GDM patients. 

Overall, our clinical study has demonstrated that dietary counseling focusing on the addition of whole berries and leafy vegetables to the diets of pregnant women with GDM results in improved glycemic control and other metabolic and inflammatory parameters. As discussed previously, there is a plethora of research examining nutritional intervention in decreasing the risk of GDM development, but there is a lack of research regarding nutritional intervention during active GDM in advanced pregnancy. This research provides unique information that has not yet been reported and is increasingly beneficial to clinical practice and management of GDM. GDM, even well-controlled, has many risks for both the mother and the fetus, including complications during delivery such as shoulder dystocia and hypoglycemia in the newborn, as well as long-lasting effects, such as increased risk of developing type 2 diabetes or other cardiovascular diseases in the newborn later in life. Thus, having nutritional interventions that are shown to significantly improve the cardiometabolic markers of GDM during pregnancy are important to hopefully decrease the risk of these complications perinatally and beyond. Additionally, all of the participants included in our study are self-reported Hispanic women, one of the racial groups at the highest risk of both development of and complications from GDM, which allows the results of our study to be applied to those who may benefit the most from the interventions highlighted in this trial [46]. Finally, because this study was a RCT by design, we are able to establish causation and highlight that the significant changes in the various dietary and metabolic markers seen in the nutrition intervention group are due to the specific interventions- increased whole berry and leafy vegetable intake. The interventions described are also safer, readily available, and likely more cost effective, than insulin or other glycemic control medications that patients may be treated with. Even if medication is required for a patient, our study has demonstrated that simple lifestyle changes can be used as an adjunct to improve the metabolic parameters associated with GDM in the mother.

However, we recognize some limitations of our study. Firstly, our intervention group was instructed to increase both whole berries and leafy vegetables, along with postprandial exercise, meaning that the significant changes in the various maternal metabolic parameters measured are the result of the combination of these different interventions. Thus, we are unable to isolate how much of a change is due to, for example, the berry intake or the postprandial exercise only, instead of the combination of these behaviors. Furthermore, the small sample size, while adequately powered for detecting differences in the various cardiometabolic and anthropometric endpoints measured, decreases the generalizability of the results to women with GDM from different ethnicities and risks, especially given that 100% of our population is from one specific ethnic group. Also, we did not measure specific blood markers of fruit and vegetable consumption, such as serum carotenoids and tocopherols. Finally, we examined outcomes in the mother, but did not examine fetal outcomes, infant birth weight and postpartum metabolic profiles. Despite these limitations, our findings fill a knowledge gap and contribute to the evidence on dietary and lifestyle interventions and their use in the management of gestational diabetes.

## 5. Conclusions

In conclusion, our randomized controlled trial has demonstrated that in women diagnosed with gestational diabetes mellitus, dietary counseling emphasizing the daily addition of whole berries and leafy vegetables during pregnancy directly modulates maternal metabolic parameters. The significant decreases in random blood glucose and serum IL-6, along with the significant increases in HDL-cholesterol and total serum antioxidant capacity, suggest that whole berry and leafy vegetable supplementation directly modulate the metabolic pathways that are implicated in gestational diabetes. Thus, given the significance of these findings and the relative ease of incorporating these dietary changes, whole berry and leafy vegetable supplementation must be incorporated in the clinical management of newly diagnosed gestational diabetes patients. These findings warrant larger studies to examine effects in mother-infant dyads for future health risks.

## Figures and Tables

**Table 1:** Baseline characteristics of women with gestational diabetes (24 to 28 weeks gestation, n = 38).

Variable	NE (n = 18)	NI (n = 20)	*p*-Value
Maternal age, y	32 ± 6	34 ± 4	0.27
Gestational age, wk	24 ± 2	26 ± 3	0.31
Body weight, lbs	205 ± 44	207 ± 35	0.43
BMI, kg/m^2^	33.4 ± 4.5	32.5 ± 2.5	0.45
Systolic blood pressure, mmHg	121 ± 10	118 ± 9	0.35
Diastolic blood pressure, mmHg	77 ± 7	80 ± 10	0.21
Triglycerides, mg/dL	162 ± 22	148 ± 17	0.21
HDL-cholesterol, mg/dL	42 ± 7	44 ± 8	0.38
Energy Intake, Kcal/d	1889 ± 650	2110 ± 486	0.23
Carbohydrates, g/d	211 ± 103	266 ± 142	0.22
Proteins, g/d	98 ± 45	108 ± 128	0.44
Total Fats, g/d	65 ± 32	57 ± 2	0.73
Fiber, g/d	15 ± 6	17 ± 6	0.43
Vitamin A–RAE, µg/d	327 ± 154	318 ± 97	0.85
Vitamin D, µg/d	18 ± 11	25 ± 22	0.38
Vitamin E, mg/d	8.1 ± 4.3	9.4 ± 4.8	0.44
Vitamin C, mg/d	57 ± 33	48 ± 32	0.48
Folate, DFE, µg/d	191 ± 71	173 ± 62	0.48
Calcium, mg/d	523 ± 269	611 ± 138	0.29
Iron, mg/d	13 ± 5	12 ± 6	0.65
Grains, % RDA met	77 ± 22	85 ± 10	0.21
Vegetables, % RDA met	38 ± 24	48 ± 12	0.14
Fruits, % RDA met	38 ± 12	27 ± 9	0.11
Dairy, % RDA met	31 ± 20	42 ± 14	0.21
Proteins, % RDA met	89 ± 24	92 ± 16	0.33
Total flavonoid intake, mg/d	38 ± 21	45 ± 18	0.21
Serum CRP, mg/L	4.1 ± 1.1	3.7 ± 0.8	0.38
Serum IL-6, pg/mL	22.5 ± 9.6	34 ± 12.8	0.23
GCT test Fasting, mg/dL 1 h, mg/dL 2 h, mg/dL	125 ± 14 188 ± 11 162 ± 8	118 ± 10 192 ± 9 172 ± 7	0.32 0.21 0.25
Glycosylated hemoglobin, %	6.2 ± 0.6	6.4 ± 0.5	0.33
Physical activity/walking, min/d	23 ± 12	17 ± 11	0.43
Total serum antioxidant capacity, µmol/L	3.6 ± 2.2	4.2 ± 3.1	0.24

Values are means ± standard deviation (SD). Significant estimates with *p* < 0.05 are shown in bold font. CRP: C-reactive protein; d: day; DFE: dietary folate equivalents; g: grams; GCT: glucose challenge test; IL-6: interleukin-6; Kcal: kilocalorie; kg: kilogram; L: liter; lbs: pounds; m: meters; mg/dL: milligrams per deciliter; mg: milligrams; min: minutes; mmHg: millimeter of mercury; n: sample size; NE: nutrition education; NI: nutrition intervention; pg: picograms; RAE: retinol activity equivalents; RDA: recommended dietary allowance; wk: week; y: years; %: percent; µg: micrograms; µmol: micromole.

**Table 2:** Dietary intakes in control and nutrition education groups between 38 to 40 weeks gestation at the end of the treatment period (n = 38).

Variable	NE (n = 18)	NI (n = 20)	*p*-Value
Energy intake, Kcal/d	1956 ± 465	2067 ± 399	0.50
Carbohydrates, g/d	243 ± 103	240 ± 120	0.97
Proteins, g/d	105 ± 40	111 ± 26	0.32
Total fats, g/d	66 ± 31	58 ± 23	0.88
Fiber, g/d	15 ± 6	23 ± 7	**0.01**
Vitamin A–RAE, µg/d	336 ± 161	368 ± 109	0.54
Vitamin D, µg/d	21 ± 18	28 ± 22	0.45
Vitamin E, mg/d	10 ± 5	14 ± 6	0.08
Vitamin C, mg/d	55 ± 28	68 ± 26	0.11
Folate, DFE, µg/d	200 ± 82	203 ± 79	0.93
Calcium, mg/d	521 ± 250	665 ± 75	0.12
Iron, mg/d	13 ± 6	11 ± 7	0.19
Grains, % RDA met	78 ± 16	82 ± 9	0.21
Vegetables, % RDA met	44 ± 19	57 ± 10	**0.04**
Fruits, % RDA met	50 ± 12	68 ± 15	**0.01**
Dairy, % RDA met	38 ± 21	45 ± 16	0.05
Proteins, % RDA met	92 ± 16	96 ± 16	0.21
Total flavonoid intake, mg/d	45 ± 14	62 ± 16	**0.01**

Values are means ± SD. Significant estimates with *p* < 0.05 are shown in bold font. d: day; DFE: dietary folate equivalents; g: grams; Kcal: kilocalorie; mg: milligrams; n: sample size; NE: nutrition education; NI: nutrition intervention; RAE: retinol activity equivalents; RDA: recommended dietary allowance; %: percent; µg: micrograms.

**Table 3:** Biochemical and behavioral changes in control and nutrition education groups between 38 to 40 weeks gestation at the end of the treatment period (n = 38).

Variable	NE (n = 18)	NI (n = 20)	*p*-Value
Body weight, lbs	212 ± 45	210 ± 25	0.67
BMI, kg/m^2^	34.4 ± 3.5	32.9 ± 1.4	0.12
Systolic blood pressure, mmHg	124 ± 8	121 ± 4	0.19
Diastolic blood pressure, mmHg	84 ± 5	78 ± 7	0.13
Triglycerides, mg/dL	173 ± 27	156 ± 16	0.21
HDL-cholesterol, mg/dL	43 ± 8	51 ± 7	**0.03**
Serum CRP, mg/L	4.2 ± 1.2	3.8 ± 0.7	0.24
Serum IL-6, pg/mL	34.6 ± 12.2	26 ± 13.8	**0.03**
Random blood glucose, mg/dL	128 ± 24	112 ± 14	**0.04**
Physical activity/walking, min/d	30 ± 12	25 ± 13	0.24
Total serum antioxidant capacity, µmol/L	3.8 ± 3.5	6.3 ± 3.8	**0.001**

Values are means ± SD. Significant estimates with *p* < 0.05 are shown in bold font. BMI: body mass index; CRP: C-reactive protein; d: day; DFE: dietary folate equivalents; g: grams; GCT: glucose challenge test; HDL: high density lipoprotein; IL-6: interleukin-6; Kcal: kilocalorie; kg: kilogram; L: liter; lbs: pounds; m: meters; mg/dL: milligrams per deciliter; mg: milligrams; min: minutes; mmHg: millimeter of mercury; n: sample size; NE: nutrition education; NI: nutrition intervention; pg: picograms; RAE: retinol activity equivalents; RDA: recommended dietary allowance; wk: week; y: years; %: percent; µg: micrograms; µmol: micromole.

## Data Availability

The research team has stored all the data in their laboratories in the Department of Kinesiology and Nutrition Sciences at UNLV in a database that is password protected.
